# Global water cycle amplifying at less than the Clausius-Clapeyron rate

**DOI:** 10.1038/srep38752

**Published:** 2016-12-09

**Authors:** Nikolaos Skliris, Jan D. Zika, George Nurser, Simon A. Josey, Robert Marsh

**Affiliations:** 1 University of Southampton, National Oceanography Centre, Southampton, UK; 2 School of Mathematics and Statistics, University of New South Wales, Sydney, Australia; 3 National Oceanography Centre, Southampton, UK

## Abstract

A change in the cycle of water from dry to wet regions of the globe would have far reaching impact on humanity. As air warms, its capacity to hold water increases at the Clausius-Clapeyron rate (CC, approximately 7% °C^−1^). Surface ocean salinity observations have suggested the water cycle has amplified at close to CC following recent global warming, a result that was found to be at odds with state-of the art climate models. Here we employ a method based on water mass transformation theory for inferring changes in the water cycle from changes in three-dimensional salinity. Using full depth salinity observations we infer a water cycle amplification of 3.0 ± 1.6% °C^−1^ over 1950–2010. Climate models agree with observations in terms of a water cycle amplification (4.3 ± 2.0% °C^−1^) substantially less than CC adding confidence to projections of total water cycle change under greenhouse gas emission scenarios.

Understanding and quantifying observed global water cycle change is key to predicting future climate^1^. The Clausius-Clapeyron relationship predicts an increase in the water holding capacity of air (the saturation water vapor pressure) of approximately 7% per degree Celsius rise in temperature^2^. It has been suggested that this would lead to a strengthening of the global evaporation (*E*) minus precipitation (*P*) pattern with global surface warming^2^,^3^, as consistently projected by state of the art climate models under anthropogenic forcing scenarios^2^,^4^. However it has not been possible to test whether the water cycle has strengthened using observations of rainfall and evaporation as accurate and comprehensive measurements are not available, particularly over the ocean^1^,^5^,^6^.

Ocean salinity is an integrator of changes in the water cycle, reflecting the exchange of fresh water between the ocean and various components of the climate system. The observed spatial pattern of the global ocean salinity multi-decadal change provides strong evidence for an amplified water cycle with highly evaporative surface subtropical regions becoming more saline and precipitation-dominated tropical and sub-polar regions becoming less saline^4^,^7^,^8^,^9^,^10^,^11^,^12^, with these features extending deep into the ocean (see Fig. S1). Observed regional salinity changes over recent decades have significantly exceeded the range of internal natural variability in control simulations of coupled climate models and have been attributed to anthropogenic forcing^13^,^14^,^15^,^16^.

A number of previous studies have inferred long-term increases in the water cycle from observed changes in the 3-D salinity field^9^,^10^,^12^. However, uncertainty in estimating global water cycle change was quite large in all these studies as calculations were based on rather strong assumptions such as zero salt fluxes between the mixed layer and the deeper ocean^9^ or advection/diffusion of salinity anomalies only along isopycnal surfaces^10^,^12^.

Durack *et al*.^4^ used an empirical method to infer global water cycle intensification from the Pattern Amplification (PA) of Sea Surface Salinity (SSS; surface being defined as the shallowest 10 m), defined as the slope of the linear regression by basin zonal averages of SSS change versus the climatological mean SSS anomaly from the global climatological SSS. The relationship between salinity PA and water cycle change in climate models from the Coupled Model Intercomparison Project Phase 3 (CMIP3) was then used to infer a water cycle (*E-P*) change from observed salinity PA changes. Their results suggested an amplification of the global water cycle of approximately 4% (8 ± 5% per degree Celsius of global mean surface temperature rise, here after °C^−1^) over 1950–2000. CMIP3 historical simulations showed considerably lower salinity PA values than observations, and also presented much less consistent results regarding the relationship between SSS patterns, water cycle change and warming.

## Research design

**Figure 1:**
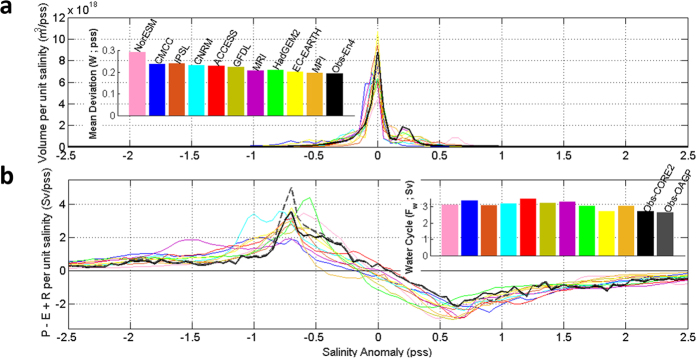
Historical mean volumetric distribution and water cycle amplitude in salinity coordinates. (**a**) Historical mean volumetric distribution of sea-water in salinity anomaly (with respect to the global mean salinity) space (m^3^/pss; colours: CMIP5; black: En4 observations); Bars show the mean deviation of salinity (*W*; pss). (**b**) Historical mean *P+R−E* in sea surface salinity anomaly space (Sv/pss; colours: as in (**a**); solid black: observations CORE2-Dai, dashed black: observations GPCP-OAFLUX-Dai). Bars show accumulated *P+R−E* up to the global mean salinity (*F*_*cycle*_; Sv).

Here we quantify recent water cycle change using a new method developed by Zika *et al*.^17^ based on water mass transformation theory^18^,^19^. Seawater can only change salinity due to the addition/removal of freshwater, i.e. through precipitation (*P*), evaporation (*E*), runoff (*R*), ice melt/freeze etc., or by mixing with waters of differing salinities. Considering the entire ocean as a volumetric distribution in salinity coordinates (Fig. 1), mixing will always draw high salinity waters towards low salinity waters collapsing the distribution towards a single value. For the distribution to be maintained, freshwater must be added to the least saline parts of the distribution and removed from the most saline parts, drawing the distribution apart. The integral of *P−E* + *R* over the salinities lower than the global mean (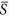) (equal and opposite to the integral over the more saline side of the distribution in a balanced system) is defined as the oceanic water cycle (*F*_*cycle*_) for the purposes of this study. It is important to note that this definition considers only the geographical redistribution of water by the atmosphere and hence patterns of *E-P.* It does not necessarily scale with changes in *P* and *E* individually as such changes can compensate leading to no change in *E-P* locally and hence no change in salinity. Zika *et al*.^17^ show that the width of the ocean’s volumetric distribution (*W*; defined as the volume weighted mean of 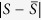) is related to the water cycle by


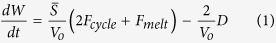


where *V*_*o*_ is the volume of the ocean. *F*_*melt*_ is the imbalance between the total freshwater added to the fresh side of the distribution and that added to the saline side (for example due to the melting of glaciers and sea-ice). To first order if an equal amount of freshwater is added to both sides *W* does not change. In practice we will assume all the additional freshwater is from melting glaciers and hence enters the ocean at polar/sub-polar latitudes where the ocean is fresher than the global mean. *D* is the diffusive flux of salt across the 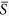 isosurface from the high salinity to the low salinity side of the distribution. The diffusive flux is thought to increase as the contrast between high and low salinity increases (i.e. as *W* increases) and hence is parameterized using an e-folding timescale *τ,* such that 2*D/V*_*0*_ = *W/τ.*

We use equation (1) to infer the water cycle change from observed changes in ocean salinity. Historical mean and trend analysis from three global gridded observational salinity datasets based on *in-situ* data covering the period 1950–2010 (CSIRO^11^, Ishii^20^, and En4^21^) are used (see Methods). Equation (1) is essentially a salt conservation equation in salinity space where salt mixing is parameterized using a simplified expression. The mixing parameterization of equation (1) is tested against output from ten state-of-the-art models from the Coupled Model Intercomparison Project Phase 5 (CMIP5)^22^ including pre-industrial, historical and Representative Concentration Pathways 4.5 (RCP4.5) and 8.5 (RCP8.5) scenarios (see Methods and Table S1).

## Results and Discussion

Figure 1a shows the historical mean volumetric distribution in salinity coordinates for the En4 observational dataset and the ten CMIP5 models considered here. Most CMIP5 models show a broader mean volumetric distribution than observations. The CMIP5 ensemble mean *W* over the historical period (1950–2005) is 0.214 ± 0.032 pss (multimodel mean ± standard deviation, see Methods) as compared to 0.192 for En4. In contrast with the large multimodel spread in *W* (~15% of the mean) *W* mean uncertainty in CMIP5 (i.e. average of individual model standard deviations of the detrended yearly timeseries) is quite small (~0.0012) and comparable to the observational uncertainty (En4; ~0.0011). The global full-depth ocean salinity distribution changes very slowly since salinity variations in the deep ocean i.e. below 1000 m, representing the major part of the total ocean volume, are very small.

Figure 1b shows the historical mean distribution of surface fresh water flux (*P* + *R−E*) in salinity coordinates for the ten CMIP5 models and for two re-analysis/satellite based air-sea flux datasets (CORE2-Dai and OAGP-Dai; see Methods). *P* + *R−E* is positive in low salinity regions and negative in high salinity regions, with the zero net fresh water flux being found near the global mean salinity value. The climatological mean water cycle amplitude (integrating *P−E* + *R* where 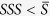 and/or *E-P-R* where 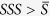) derived from observations is 2.65 ± 0.23 Sv (1 Sv = 10^6^ m^3^ s^−1^) for OAGP-Dai and 2.70 ± 0.25 Sv for CORE2-Dai (see Methods). The CMIP5 ensemble mean water cycle amplitude averaged over the historical period (1950–2005) amounts to 3.24 ± 0.28 Sv (multimodel mean + standard deviation), which is substantially larger than the two observationally-based estimates. The broader salinity volumetric distribution in CMIP5 models is likely to reflect their higher water cycle strength as compared to observations.

Applying a steady state assumption to equation (1) and using the model 2*D/V*_*0*_ = *W/*τ we previously reported a mixing timescale τ ≈ 48.3 ± 4.6 years^17^ from the En3 observational analysis (this result is identical for En4). We calculate τ ≈ 42.4 ± 6.8 years for the CMIP5 pre-industrial ensemble mean.

**Figure 2:**
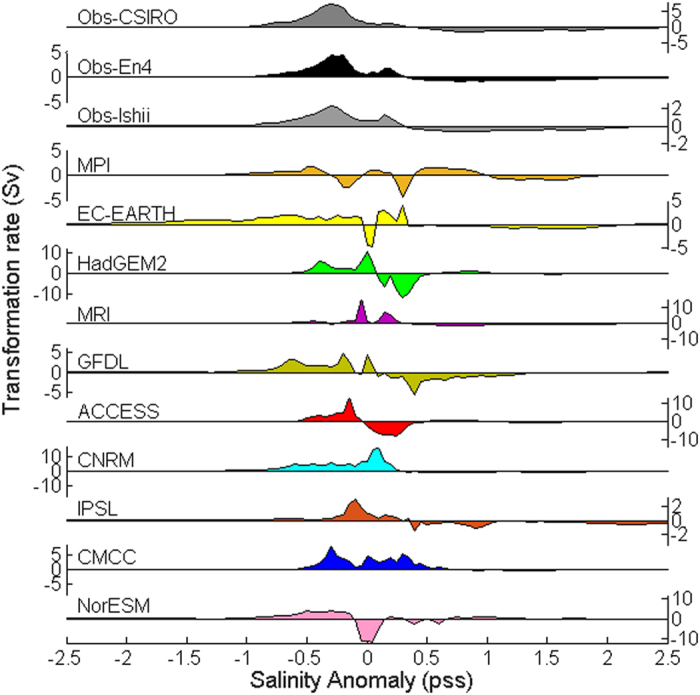
Rate of change of volume accumulated in salinity coordinates. Rate of change of volume accumulated in salinity coordinates from low salinity to high salinity (Sv), equivalent to a transformation rate. Salinity anomaly is defined with respect to global mean salinity. Positive values represent an accumulation of less saline water (transformation from saline to fresh water) and negative values represent an accumulation of more saline water (transformation from fresh to saline water). Shown are the three observational datasets (over 1950–2010) and the ten CMIP5 historical simulations (over 1950–2005) (Colours: as in Fig. 1). Positive transformation rates mainly occur in the fresh regions and negative transformation rates mainly occur in the saline regions.

All observational products show an amplification of the mean 3-dimensional salinity field over 1950–2010 (See Supplementary Fig. 1). In general isohaline layers become thicker in both low-salinity and high-salinity regions, while thinning at salinities around the global mean, with the fresh Southern Ocean showing the larger isohaline volume increases. High and low salinity water is formed at the expense of salinity classes closer to the mean (Fig. 2). As the water cycle amplifies high-salinity waters are transformed into more saline waters in dry (evaporation-dominated) regions, and low-salinity waters are transformed into less saline waters in fresh (precipitation-dominated) regions. Fresh regions become fresher and saline regions become more saline consistent with the “wet gets wetter, dry gets drier” paradigm^23^. This suggests a freshwater displacement in salinity space, i.e. from high-salinity to low-salinity regions. There are substantial differences in the resulting change of the salinity volumetric distribution between CMIP5 models as well as between models and observations (see Fig. 2). However, most models, in accordance with observations, show a significant stretching of the salinity distribution as the water cycle amplifies, with mostly positive freshwater transformation rates representing an accumulation of less saline water in the fresh regions (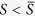) and mostly negative transformation rates representing an accumulation of more saline water in the saline regions (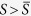).

This stretching of the salinity distribution results in an increase in the mean deviation (*W’;* change in *W* over time) of 0.0035 pss (En4), 0.0020 pss (Ishii), 0.0045 pss (CSIRO), and 0.0034 ± 0.0020 pss (CMIP5) over the historical period. The changes in *W* observed are larger than the standard deviation of *W* in En4 but are small (e.g. of the order of the precision of typical salinometers) since they combine order 0.5 pss changes in salinity near the surface with vanishingly small signals in the deep ocean (see Supplementary Fig. 1). Estimates of *W ′* are substantially larger when only the upper ocean is considered (e.g. *W*′ = 0.015pss above 1000 m from En4 data and by which depth 80% of the signal in water cycle change signal is detected; Supplementary Fig. 2).

All observational datasets indicate that the volumetric distribution shifts toward lower salinities, i.e. on average, fresh regions become fresher at a higher rate than saline regions become more saline (see Fig. 2 and supplementary Fig. 1). This suggests a global net freshwater input. Considering the change in global mean salinity, the apparent net freshwater input is *F*_*melt*_ = 0.029 Sv for En4, 0.025 Sv for Ishii, and 0.044 Sv for CSIRO.

Given both the water cycle change and the salinity distribution change are known for the CMIP5 ensemble we can use these to test equation (1). We should stress here that we do not use CMIP5 data to calibrate any parameters in (1); we only use them to test the robustness of its mixing parameterization.

**Figure 3:**
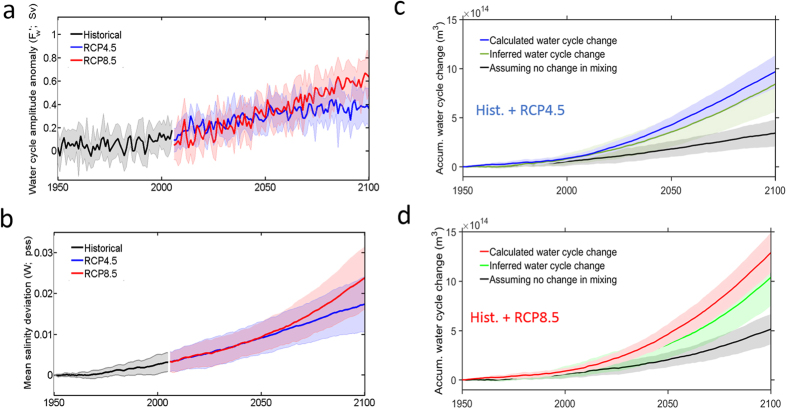
Accumulated water cycle change versus change in salinity volumetric distribution in CMIP5 simulations. (**a**) Water cycle amplitude anomaly (*F*_*cycle*_’; anomaly with respect to the pre-industrial mean) in the CMIP5 historical (1950–2005; black line), RCP4.5 (2006–2100; blue line), and RCP8.5 (2006–2100; red line) simulations. (**b**) Mean salinity deviation anomaly (*W ′*; anomaly with respect to 1950) in historical (1950–2005; black line), RCP4.5 (2006–2100; blue line) and RCP8.5 (2006–2100; red line) simulations. (**c**) Accumulated fresh water transport from high salinity to low salinity regions by the water cycle 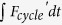 (calculated: blue lines; inferred from equation (1): green lines; inferred from equation (1) assuming no change in mixing: black lines) for the historical and RCP4.5 scenario ensemble mean (1950–2100). (**d**) same as (**c**) but for RCP8.5. Solid lines depict ensemble means and shaded areas depict one standard deviation limits.

Variations of the water cycle amplitude (*F*_*cycle*_) in CMIP5 show large inter-annual variability but also large, statistically significant (at the 95% confidence interval) positive linear trends over the 21^st^ century in all RCP4.5 and RCP8.5 model runs (Fig. 3a). In most models the water cycle increases close to linearly from the late 1970 s to the end of 21st century. Considering the CMIP5 ensemble means, the water cycle amplification rate (with respect to the pre-industrial mean) is estimated to be 2.4 ± 1.7% (4.0 ± 3.0%/°C) in the historical, 7.2 ± 2.4% (4.4 ± 1.5%/°C) in the RCP4.5, and 18.2 ± 4.5% (4.6 ± 1.3%/°C) in the RCP8.5 runs, respectively.

The ensemble mean for the total change in *W* over 2006–2100 is 0.0121 ± 0.0031 pss for the RCP4.5 and 0.0192 ± 0.0042 pss for the RCP8.5 scenario runs respectively. In all CMIP5 RCP4.5/8.5 scenario runs *W* gradually increases over the 21^st^ century (Fig. 3b).

Figure 3c,d show the accumulated integral (in time) of the *F*_*cycle*_ anomaly with respect to 1950. Black lines show the accumulated water cycle change that would be predicted by equation (1) if the diffusive flux (*D*) did not change. As the water cycle amplifies, the width of the salinity distribution increases and the salinity contrast between water masses becomes larger, resulting in an increasing diffusive salt flux that damps the growth in width of the salinity distribution. Considering the CMIP5 ensemble mean for the RCP4.5 and RCP8.5 runs (1950–2100), the progressive increase in salt mixing over that period reduces the net stretching of the salinity distribution (i.e. driven by the water cycle increase) by approximately 60% at the end of 21^st^ century.

Given mixing timescales derived from the pre-industrial states of each CMIP5 model and the model’s changes in *W* and *F*_*melt*_, we solve equation (1) to infer the accumulated change in *F*_*cycle*_ (Fig. 3c,d). The calculated water cycle change from changes in model *E, P*, and *R* compares favorably with that inferred from equation (1) using model 3-D salinity change). The ensemble mean of the calculated water cycle change falls within 1 standard deviation of the inferred values for both scenarios and for all years – although the ensemble mean of the inferred change tends to under-predict the ensemble mean of the calculated change.

**Figure 4:**
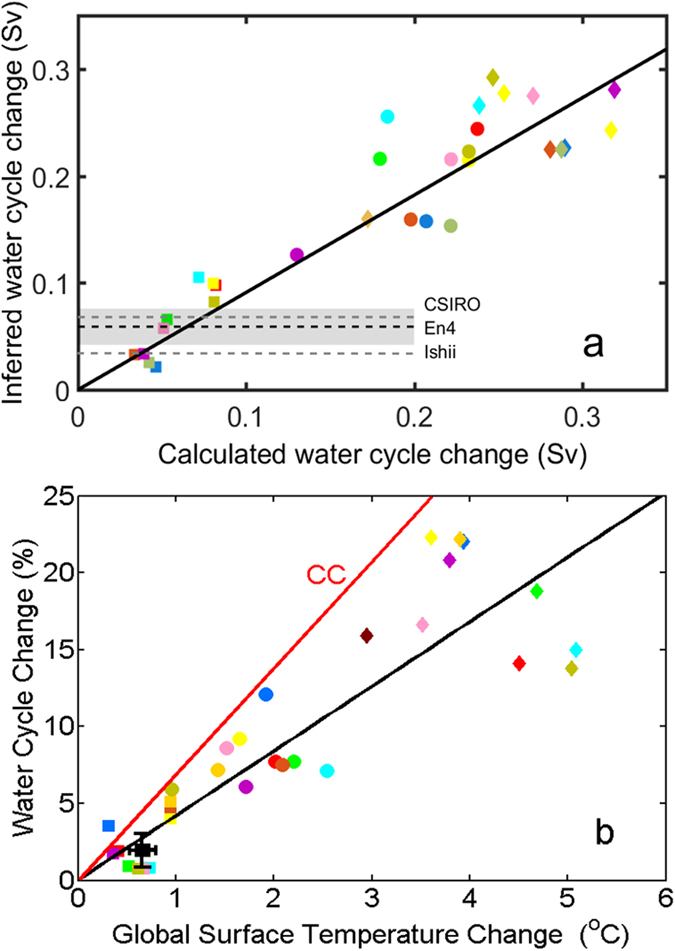
Calculated versus inferred water cycle change in CMIP5 models. (**a**) Calculated water cycle change versus inferred water cycle change using equation (1) based on changes in the mean salinity deviation. Horizontal dashed lines show the water cycle change inferred from the three observational estimates. Grey shaded area depicts one standard deviation limits for En4. Also shown is the line of best fit (black line; slope 90%; r = 0.92; p < 0.01). (**b**) Change in water cycle as a percentage of historical mean versus change in global mean surface temperature. Also shown are lines of best fit (black; slope 4.2%/°C; r = 0.87; p < 0.01) and of CC rate (red; 7%/°C). Squares show historical, circles show RCP4.5, and diamonds show RCP8.5 simulations (colours correspond to models as in Fig. 1). Black square with uncertainty range depicts the mean from the three observational estimates.

Individual model-calculated water cycle changes are compared with changes inferred from equation (1) in each case using the mixing timescale appropriate to that model (Fig. 4a). This shows a robust linear relationship between the calculated and inferred water cycle change (R = 0.92, statistically significant at the 99% confidence interval). The slope of the one-to-one line is indistinguishable from the line of best fit in terms of correlation suggesting there is no consistent bias in the method. Standard deviation of inferred values with respect to the one-to-one line is ~22% of the mean change. This provides an estimate of the uncertainty of our method (i.e. using parameterized equation (1) to infer water cycle change).

We have now established that equation (1), with mixing timescales inferred from historical/pre-industrial balances, represents a robust relationship between water cycle change and salinity distribution change. Using observational estimates of *W ′* (assuming a linear trend), *F*_*melt*_ and *τ,* and combining the uncertainties arising from these data with the method uncertainty (see Methods) we estimate a change in *F*_*cycle*_ of 0.059 ± 0.022 Sv for En4, 0.032 ± 0.012 Sv for Ishii, and 0.068 ± 0.025 Sv for CSIRO between 1950 and 2010. Considering the climatological mean water cycle obtained from the re-analysis/observational data, these changes imply an amplification of the water cycle (i.e. defined as the water cycle change inferred from equation (1) divided by the climatological mean water cycle amplitude) of 1.19 ± 0.46% for Ishii, 2.21 ± 0.85% for En4 and 2.54 ± 0.96% for CSIRO over 1950–2010.

Figure 4b shows the change in water cycle amplitude versus the global-mean surface air temperature change for the ten CMIP5 models in their historical (1950–2005), RCP4.5 (2006–2100), and RCP8.5 (2006–2100) runs. Results indicate a robust relationship between increasing water cycle amplitude and surface warming. The linear trend (R~0.87, statistically significant at the 99% confidence interval) gives a water cycle amplitude sensitivity to warming of 4.3 ± 2.0%/°C for CMIP5, which is much less than that predicted by the Clausius-Clapeyron relationship (~7%^/^°C). The observationally inferred water cycle change versus global surface temperature change over 1950–2010 (0.66 ± 0.13 °C, from HadCRUT4^24^) with a slope of 1.80 ± 0.78% °C^−1^ for Ishii, 3.35 ± 1.44% °C^−1^ for En4 and 3.85 ± 1.64% °C^−1^ for CSIRO, sits within the spread of CMIP5 models (4.3 ± 2.0% °C^−1^). Both models and observations are consistent in terms of their oceanic water cycle response to a given amount of global surface warming, within the large uncertainties of each.

Our estimate of water cycle sensitivity to warming based on our CMIP5 ensemble (~4.3%/°C) is very close to the estimate of Durack *et al*.^4^ based on the CMIP3 ensemble of E-P PA (~4.5%/°C). It is interesting to note that in climate models total *P* is increasing at a significantly lower rates (~3.4%/°C, Allen and Ingram^3^; ~2%/°C, Held and Soden^2^; 3.1%/°C, Durack *et al*.^4^ - than the global E-P pattern (~4.5%/°C, Durack *et al*.^4^). Although precipitation extremes are shown to follow CC scaling, the total global average precipitation, commonly referred to as the strength of the water cycle in climate sciences, is constrained by the atmospheric radiative energy balance (e.g. Allen and Ingram^3^, Held and Soden^2^). A change in the water cycle well below the CC rate was exhibited by the majority of coupled climate models (Allen and Ingram^3^; Held and Soden^2^; Durack *et al*.^4^).

Using salinity observations we infer a water cycle amplification of 3.0 ± 1.6%/°C, which is approximately half the CC rate. A lower increase in the water cycle than the CC rate has been attributed to a weakening of the atmospheric circulation due to warming, leading to a partial compensation for the water cycle increase (e.g., Held and Soden^2^). Our observational salinity-based estimate of water cycle change is substantially smaller than that reported by Durack *et al*.^4^ (~8 ± 5%/°C). Our approach differs from Durack *et al*.^4^ in that it is based on a salt conservation law, linking water cycle change to ocean salinity rather than an empirical relationship. In addition, our method exploits the full 3-D salinity observations. Differences between observed and modelled surface salinity changes as highlighted by Durack *et al*.^4^ may, for example, reflect biases in the way models simulate the ventilation of surface signals into the ocean interior^25^. Another important difference is that our analysis distinguishes between water cycle change (i.e. associated with the increase in the salinity contrast between water masses) and global net fresh water input (*F*_*melt*_). All datasets show a large global net freshening amounting to ~0.044 Sv for CSIRO, ~0.025 Sv for Ishii and ~0.029 Sv for En4 that is of the same order of magnitude with the water cycle increase (i.e. ~50–80% of *F*_*cycle*_’). If the *F*_*melt*_ term is not included in the calculation we infer much larger water cycle changes of 0.048 ± 0.019 Sv for Ishii, 0.073 ± 0.028 Sv for En4, and 0.097 ± 0.037 Sv for CSIRO. This effect alone would bring the CSIRO estimate of water cycle change up to ~5.5 ± 2.3% °C^−1^. Although CMIP5 models do not have interactive ice sheets some models do show net freshening due to small unbalances in their freshwater budget. However the ensemble mean of net freshening (0.11 ± 0.21 Sv) is small and not statistically significant. When the net freshening is not accounted for in (1; *F*_*melt*_  = 0) the correlation and slope between the calculated water cycle change and that inferred using equation (1) reduces to 0.86 (from 0.92) and 83% (from 90%), respectively. The attribution by Durack *et al*.^4^ of all surface salinity contrast change to the water cycle, rather than in part to a net addition of freshwater, may help explain the large water cycle change they infer.

The global net freshening estimated by the three observational products could be due to a real physical change or a general bias in the observational analysis. A source of bias could be the ill-conditioning of salinity trends due to the lack of observations over earlier decades and in the Southern Hemisphere and deep layers^11^.

An actual input of extra fresh water is plausible especially at high-latitudes due to glacial and sea-ice melt. An equivalent fresh water input due to the observed 2 mm/yr (3.2 mm/yr) global sea level rise over 1971–2010 (1993–2010; satellite data only) mainly attributed to glacier melt and thermal expansion amounts to 0.024 Sv (0.038 Sv)^25^. If we remove the estimated thermal expansion contribution to sea level rise for this period of 40% (34% for satellite only period)^26^ we obtain a value of ~0.015 Sv (0.023 Sv), which is of the same order of magnitude to that inferred from the net freshening of the EN4 and Ishii datasets.

CMIP5 outputs have been used here to test (not calibrate) the mixing parameterization of our method, and, in particular, to confirm that in physically consistent representations of the global ocean, mixing effects which control the mean salinity distribution also control its response to forcing changes. There may be an asymmetry between the mixing effects which set the mean state and those which arise in response to transient climate change that is not captured in either our theory or any of the climate models considered, which is an important caveat of this work. There are large uncertainties due to different representations of processes, and different mixing parameterizations in particular, in the various CMIP5 models. This results in a quite large multimodel standard deviation for the inferred water cycle change from equation (1) (~50% of the mean). However, although climate model simulations for the same period display a diverse range of solutions in terms of where salinity changes occur, they agree with observations, within the large uncertainties of each, in terms of their water cycle response to global warming.

Another large source of uncertainty in our analysis is the spread in the water cycle amplification inferred from the three observational salinity datasets. Although these datasets are based on common *in situ* observations, they use different trend analysis methods and space/time interpolation techniques to deal with the large data gaps. Uncertainties of our observationally-based estimates are quite large (~40% of the mean) but generally lower than those of previously reported estimates of global water cycle change inferred from observed salinity change using different methods - i.e. Durack *et al*.^4^ infer 8 ± 5%/°C over 1950–2000, while Skliris *et al*.^12^ infer 4.7 ± 2.5%/°C over 1950–2010.

Our estimates of the magnitude of water cycle change do not rely on the accuracy of CMIP5. However we do rely on CMIP5, in part, for our uncertainty estimates. If the relationship between the range of possible water cycle changes and the range of salinity distribution changes in CMIP5 were to be an underestimate (over-estimate) this would lead us to underestimate (overestimate) our water cycle change uncertainty. Our uncertainty estimate also relies on the range of salinity distribution change estimates from observations being representative of the range of possible actual changes. If the range of changes were under-estimated (over-estimated) by the observational network and the mapping techniques used to produce the three data sets, this would also lead to an underestimate (over-estimate) of the uncertainty. Although we cannot quantify how these factors contribute to the uncertainty it is our opinion that they are more likely to lead to larger than smaller uncertainties.

Data sparsity certainly limits the applicability of this method over the earlier decades of the observational record. However this method is expected to become progressively more applicable as salinity sampling increases. The continuous acquisition of 3-D salinity data by the ARGO network since the early 2000 s, achieving an unprecedented near-global ocean observation, will help us to progressively enhance the present knowledge of changing salinity and the water cycle.

Despite the sources of uncertainty stated above, the water cycle amplification we estimate from salinity observations (3.0 ± 1.6% °C^−1^) is well within the spread of climate model projections (4.3 ± 2.0% °C^−1^). This adds confidence to projections of global water cycle amplification substantially below the CC rate under anthropogenic greenhouse gas emission scenarios.

## Methods

### Total water displaced from saline to fresh regions by the water cycle

Equation (1) is equivalent to equation (A13) of Zika *et al*.^17^ where the change in global mean salinity is moved to the right hand side and converted into a term proportional to the freshwater input *F*_*melt*_. Applying the model 2*D/V*_*0*_  = *W/τ* and integrating (1) in time shows the relationship between the change over time in width of the salinity distribution (*W’*) and the total water displaced from saline to fresh regions by the water cycle (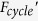):


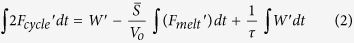


where *W´,* 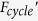, and *F*_*melt*_*´* are deviations from a long term mean in steady state. In order to solve for 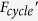 we assume it varies linearly with time.

### CMIP5 model simulations

We analysed the output of the pre-industrial, historical, Representative Concentration Pathways 4.5 (RCP4.5) and 8.5 (RCP8.5) simulations from ten Coupled Model Intercomparison Project Phase 5 (CMIP5)^22^ models including ACCESS1.3, CMCC-CM, CNRM-CM5, GFDL-ESM2M, HadGEM2-ES, IPSL-CM5A-LR, MPI-ESM-MR, MRI-CGCM3, EC-EARTH, NorESM1-M. The model selection is based on the output availability of three variables: 3-D salinity, net surface fresh water flux (precipitation + river runoff + ice melting - evaporation - ice formation), and surface air temperature. From the NCAS British Atmospheric Data Centre^27^ we retrieved monthly 3-D salinity (so), monthly net surface fresh water flux (wfo), as well as monthly surface air temperature (tas) fields. The periods analysed include a 150-year period from the pre-industrial simulations, 1950–2005 from the historical simulations, and 2006–2100 from the RCP4.5, and RCP8.5 simulations. All models are tested in their pre-industrial control runs and they do not show any statistical significant trend in the width of their salinity volumetric distribution or in their water cycle.

### Data sources

#### Salinity observational data

The global salinity change over 1950–2010 is assessed using three observational datasets a) the UK Met Office Hadley Centre Enhanced Ocean Data Assimilation and Climate prediction (ENACT) archive version4 (En4, subversion En4.1.1, 1° x 1° grid) dataset (http://www.metoffice.gov.uk/hadobs/en4) developed by Good *et al*.^21^, b) the Ishii (Ishii, subversion v6.13, 1°x1° grid) dataset (http://www.data.jma.go.jp/gmd/kaiyou/english/ohc/ohc_data_en.html) developed by Ishii and Kimoto^20^, and c) the CSIRO dataset (1° x 2° grid) (http://www.cmar.csiro.au/oceanchange) developed by Durack and Wijffels^11^. All three datasets consist of quality-controlled temperature and salinity (PSS-78) profiles spanning the period 1950–2010 with most of original data sources being common including the World Ocean Database (2005), the Global Temperature-Salinity Profile Programme (from 1990) and profiling float data from the Argo Global Data Assembly Center (from 1999). The datasets have significant differences in their trend analysis methodology. Unlike the En4 and Ishii datasets for which analysis is based on objectively-analyzed monthly fields, the CSIRO dataset uses a variable search radius, dependent on spatial and temporal sampling, which leads to highly-variable “spatial footprints” of mapped trends in the pre-Argo era so that broad-scale trends are appropriately emphasized. While in the En4 dataset trends are linearly fitted to the interpolated annual mean data provided by the objectively-analysed monthly fields^12^, in CSIRO dataset the salinity trends are fitted concurrently with the mean climatology and the ENSO signal through a multiple linear regression^11^. Another difference between the datasets concerns their depth/area coverage. While the trend analysis in the En4 dataset involves the global ocean, in the Ishii (CSIRO) dataset only the upper 0–3000 m (0–2000 m) layer is considered. Moreover in the CSIRO dataset all marginal seas and regions north (south) of 70°N (°S) are excluded from the trend analysis. When we consider the same smaller area/depth coverage for En4 as for CSIRO dataset, we obtain slightly lower values for both the water cycle increase (~10%) and the global net freshening (~20%). On the other hand, all datasets have almost identical climatological mean volumetric distribution in salinity space when considering the common smaller area/depth coverage. Assuming changes in the deep ocean not too different in the three datasets, we are using the En4 deep ocean data to extrapolate the Ishii and CSIRO-derived values for the water cycle increase for the global ocean area/depth coverage.

#### Re-analysis/observational P+R−E data

Two combined atmospheric reanalysis/observational global ocean *E-P-R* datasets are included in the present study to assess the climatological mean global freshwater cycle in salinity space as described in Zika *et al*.^17^.

- A hybrid product spanning the period 1979–2010 in which *E* is provided by the Objectively Analyzed air-sea Fluxes (OAFlux) dataset (http://rda.ucar.edu/datasets/ds260.1/)^28^, that blends NCEP and ERA-40 reanalysis products with satellite surface meteorology through an objective synthesis, *P* is obtained from the Global Precipitation Climatology Project (GPCP v2.2, http://rda.ucar.edu/datasets/ds728.2/)^29^ and *R* is based on recent estimates from Dai *et al*.^30^ (OAGP-Dai thereafter). *R* includes river runoff which gives a global mean river fresh water discharge of 1.18 Sv^30^, and the ice melting flux from Antarctica (0.06 Sv) and Greenland (0.01 Sv) resulting in a total fresh water discharge of 1.25 Sv^6^.
- The Common Ocean-ice Reference Experiments 2 (COREv2) E-P dataset (http://rda.ucar.edu/datasets/ds260.2/)^31^, spanning the period 1979–2006 combined with the *R* product described above (CORE2-Dai thereafter). The E field is based on the NCEP/NCAR reanalysis with various adjustments and the *P* field is a blend of precipitation products including GPCP and the CPC Merged Analysis of Precipitation (CMAP)^32^ datasets, both based on rain gauge observations and satellite retrievals.

If the global annual mean fresh water budget is balanced, the accumulated integral of *P+R−E* in salinity space should sum to 0 at the end of integration. For the OAGP-Dai dataset a value of 0.45 ± 0.51 Sv is obtained indicative of a significant net freshening (*E* < *P* + *R*) while for CORE2-Dai a value of 0.05 ± 0.15 Sv is obtained showing again a net freshening but with a global fresh water budget closer to balanced. CMIP5 models considered here are very close to balanced at their pre-industrial state (not shown) whilst most of them show a small global net freshening over the historical period with the ensemble mean value being 0.11 ± 0.21 Sv.

### Uncertainty estimates and trends

Uncertainties stated concerning CMIP5 ensemble means are the standard deviation of the ensemble (multimodel mean). Uncertainties stated concerning temporal means from observational data (i.e. global spatial patterns of atmospheric fluxes, temperature, and salinity) or CMIP5 mean uncertainties (i.e. average of individual model uncertainties) are the standard deviation of the yearly detrended timeseries and uncertainties in linear trends are the standard error of a linear fit (least squares). Although CMIP5 results are not used to calibrate the method, CMIP5 spread is used to estimate the method uncertainty i.e. from the standard deviation of inferred versus calculated water cycle change in Fig. 4a. Total uncertainty in *F*_*cycle*_ change for the three observational datasets is calculated as the root mean square of the method uncertainty and the data uncertainty in equation (1), which is consistent in the two types of uncertainty are normal. Data uncertainty is derived from the standard error of the linear fit of *W* annual timeseries and the standard deviation of the mixing timescale *τ* temporal mean. Data uncertainty in the three observational datasets (~30–32% of the mean water change) is higher than the method uncertainty (~22% of the mean water cycle change). Where a single observationally based estimate of water cycle change is stated it is the arithmetic mean of estimates based on the three datasets and the uncertainty is the root mean square of their uncertainties. In most models the water cycle amplitude increases very close to linearly from the late 1970 s to the end of 21st century following the global surface warming. A more sophisticated fit taking into account the exponential nature of the CC relationship^33^ could be used but since trends are small and uncertainties are quite large for simplicity a linear fit is applied.

### Water cycle amplification rates

Water cycle amplification rate (%) is defined as the water cycle amplitude change (inferred from equation (1) for the observational estimates) divided by the climatological mean water cycle amplitude (i.e. from long-term mean distribution of *P−E+R* in observations and historical CMIP5 simulations). We also express water cycle change as a water cycle sensitivity to global surface warming (%/°C; amplification (%) per degree of global mean surface temperature rise (/°C)) to compare our results with the CC relationship.

## Additional Information

**How to cite this article**: Skliris, N. *et al*. Global water cycle amplifying at less than the Clausius-Clapeyron rate. *Sci. Rep.* **6**, 38752; doi: 10.1038/srep38752 (2016).

**Publisher's note:** Springer Nature remains neutral with regard to jurisdictional claims in published maps and institutional affiliations.

## Supplementary Material

Supplementary Information
