# Supplementary material for: Global water cycle amplifying at less than the Clausius-Clapeyron rate
Source: Sci Rep. 2016 Dec 9;6:38752. doi: 10.1038/srep38752 (PMC5146653; doi:10.1038/srep38752)
Supplement: Supplementary Information [file srep38752-s1.pdf]

Global water cycle amplifying at less than the Clausius-Clapeyron rate

Nikolaos Skliris[1,*,†], Jan D. Zika[2,†], George Nurser[3], Simon A. Josey[3], Robert Marsh[1].

[1]University of Southampton, National Oceanography Centre, Southampton, UK

[2]Department of Physics and Grantham Institute Climate Change and the Environment, Imperial College London, UK

[3]National Oceanography Centre, Southampton, UK

*Correspondence to: N.Skliris@noc.soton.ac.uk (N.S.).
† These authors contributed equally to this work

Supplementary Results

Figure S1

Figure S1shows the salinity change over 1950-2010 in a global zonally-averaged section inferred from the three observational salinity datasets. We should stress here that in our calculations inferring water cycle change the full three-dimensional field of salinity is used. Although all datasets show an amplification of the 3-D salinity field, there are differences between the products both in terms of magnitude and pattern, particularly in the Northern Hemisphere where freshening in one ocean basin can counteract salinification in another. In general, multi-decadal salinity anomalies in both surface and intermediate/deep layers are larger in CSIRO than in En4 and Ishii datasets. This is particularly so in areas of poor observational spatiotemporal coverage such as the Southern Ocean where Ishii and En4 trend analysis probably underestimates salinity anomalies as opposed to the CSIRO methodology which focuses on broad-scale footprints[11, 12].

Supplementary tables

Table S1. Changes in the salinity volumetric distribution and water cycle amplitude in CMIP5 simulations. The mixing timescale (τ), width of the salinity volumetric distribution

(W), and water cycle amplitude (F_{cycle}) in the 10 CMIP5 models considered in this study. Pre-industrial means and total changes based on linear trends over the historical (1950-2005), RCP4.5 and RCP8.5 (2006-2100) simulations are shown. Observational estimates are also shown for the mean and change in salinity distribution (En4, 1950-2010) and for the mean water cycle amplitude (OAGP-Dai, 1979-2010; CORE2-Dai, 1979-2006).

	**τ mean (yrs)**	**W mean (pss)**	**ΔW historic (pss)**	**ΔW RCP4.5 (pss)**	**ΔW RCP8.5 (pss)**	**F_{cycle} mean (Sv)**	**ΔF_{cycle} historic (%)**	**ΔF_{cycle} RCP4.5 (%)**	**ΔF_{cycle} RCP8.5 (%)**
ACCESS1.3	40.1	0.224	0.0061	0.0189	0.0259	3.60	0.8	7.1	15.0
CMCC-CM	41.3	0.243	0.0009	0.0097	0.0185	3.37	3.5	12.1	22.0
CNRM-CM5	44.6	0.227	0.0053	0.0136	0.0153	3.20	0.8	8.6	16.6
GFDL-ESM2M	43.1	0.223	0.0050	0.0185	0.0227	3.31	4.7	13.9	19.9
HadGEM2-ES	41.2	0.201	0.0042	0.0162	0.0250	3.53	0.8	5.9	15.9
IPSL-CM5A-LR	38.3	0.205	0.0039	0.0109	0.0169	3.07	4.0	9.2	22.3
MPI-ESM-MR	35.3	0.239	0.0024	0.0081	0.0189	3.03	5.1	7.2	22.2
MRI-CGCM3	35.8	0.196	0.0010	0.0069	0.0086	3.50	1.9	4.3	14.1
EC-EARTH	45.7	0.208	0.0019	0.0137	0.0187	2.70	1.7	6.1	20.8
NorESM1-M	59.1	0.293	0.0032	0.0230	0.0327	3.09	0.9	7.7	18.8
CMIP5 ensemble	42.4 ±6.8	0.226 ±0.028	0.0034 ±0.0018	0.0139 ±0.0051	0.0203 ±0.0066	3.24 ±0.28	2.4 ±1.7	7.2 ±2.4	18.2 ±4.5
En4	48.3 ±4.6	0.192 ±0.0011	0.0035 ±0.0009						
OAGP-Dai						2.65 ±0.23			
CORE2-Dai						2.70 ±0.25			

Supplementary Figures

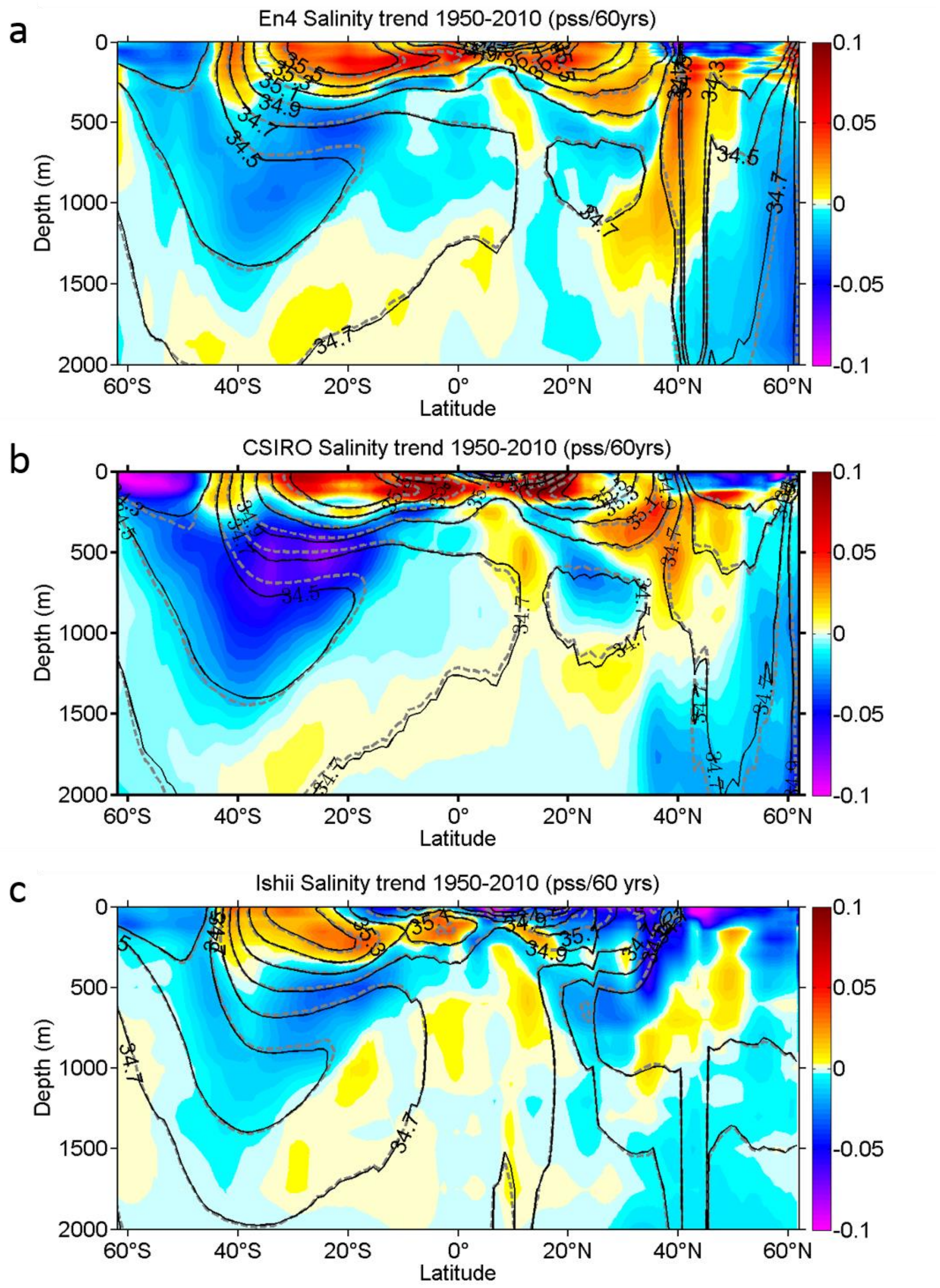

Figure S1: Observed global ocean salinity changes over 1950-2010.

Upper ocean (0-2000m depth) global zonally-averaged salinity linear trend (pss/60 yrs) over 1950-2010 for (a) En4, (b) CSIRO, and (c) Ishii. Marginal seas and areas where the sea floor is shallower than 1000m depth are excluded. Contours show global zonally-averaged salinity for years 1950 (black) and 2010 (grey dashed).

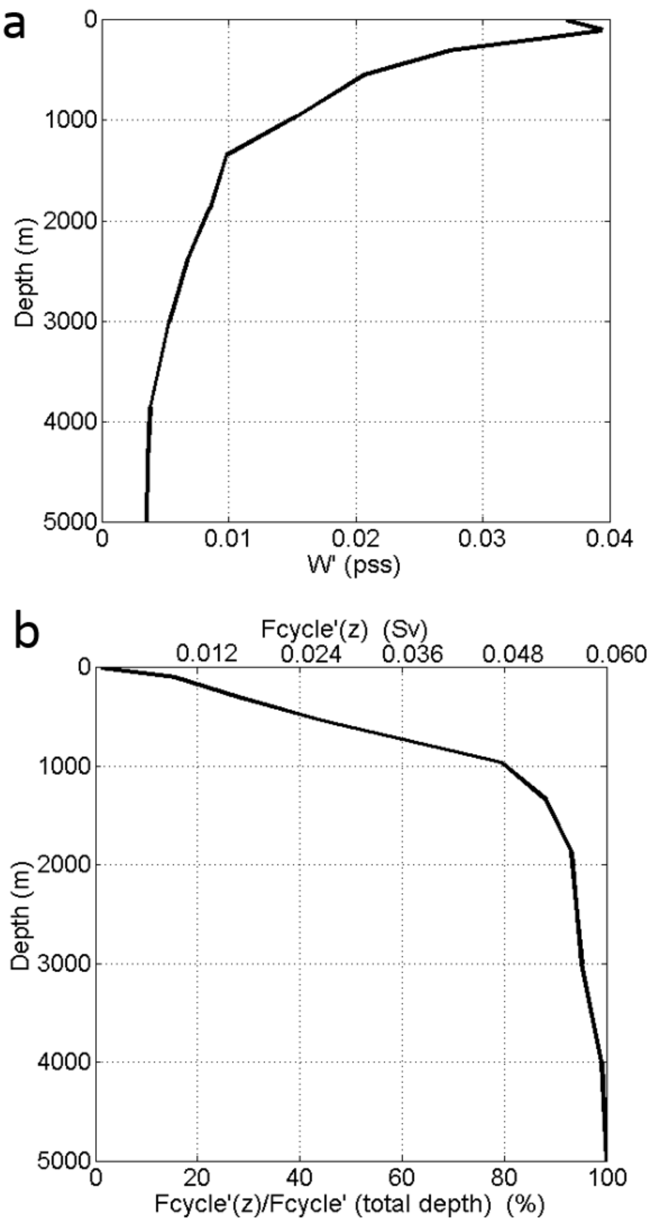

Figure S2: Observed changes in the salinity volumetric distribution and water cycle amplitude as a function of depth considered.

(a) Change in mean deviation $W'(z)$ derived from En4 data shallower than depth z. (b) $F_{cycle}'(z)$ derived using equation (1) using $W'(z)$ from data shallower than depth z and V_0 representative of the total volume of sea water shallower than z. The bottom axis shows the percentage of the total F_{cycle}' estimated up to each depth.
